# Designing teacher professional development programs to support a rapid shift to digital

**DOI:** 10.1007/s11423-020-09863-5

**Published:** 2020-11-17

**Authors:** Tania Heap, Ruthanne Thompson, Adam Fein

**Affiliations:** Division of Digital Strategy and Innovation (DSI), University of North Texas, Denton, TX USA

**Keywords:** Faculty professional development, Online teaching, Blended teaching

## Abstract

From a design perspective, this paper offers a response to the impact, value, and application of a manuscript published by Philipsen et al. (Improving teacher professional development for online and blended learning: A systematic meta-aggregative review. *Educational Technology and Research Development, 67*, 1145–1174. 10.1007/s11423-019-09645-8, 2019). Philipsen et al. (2019) reviewed what constitutes an effective teacher professional development program (TPD) for online and blended learning (OBL), with our response focusing on its value and application in light of an emergency shift to digital to address a global pandemic. This paper also proceeds to examine limitations in previous research into the subject and future research opportunities to investigate important components that inform the design of a resilient and scalable TPD for OBL.

## Impact/value

The paper presents the findings of a meta-aggregated analysis of 15 peer-reviewed articles focused on teacher professional development (TPD) programs for online and blended learning (OBL). Results from this analysis led to identification of synthesized findings on the most valuable elements of a TPD for OBL.

In “shifting to digital”, Philipsen et al.’s (2019) findings offer insights into the design and development of curricula that help teachers become successful in online environments. Among the categories identified by the authors that most impact a shift to digital are support and feedback from learning technologists, financial sustainability, program goals and procedures, changes in teachers’ skills and attitudes, relevance and evaluation of the TPD program, and support among institutional leadership.

While the authors note the importance of careful planning in  creating successful TPD for OBL, the rapid shift to digital in the spring of 2020 due to campus closures did not allow for extensive resource planning (Zhu and Liu 2020). The rush to online created by COVID-19 meant that many of the best practices for online learning and training on learning technologies were set aside in favor of emergency training on the basics of online course delivery (Buckley 2020).

Despite the challenges brought by a pandemic, Philipsen et al.’s (2019) meta-aggregated analysis offers a conceptual framework of key components and categories of an effective TPD for OBL that can be used as a lens for teacher professional developers to create and build upon new TPB initiatives. Indeed, in their paper, Philipsen and colleagues encourage their audience to expand on their research in order to refine the TPD framework.

## Application

In preparing teachers for online instruction, Philipsen et al. (2019) note the importance of support and feedback from learning designers and technologists, of offering examples of good online teaching strategies, and of enabling former TPD cohort members to maintain access to TPD materials, allowing them to refer to them as needed in their practice. A faculty development program for medical educators in a Canadian school (Buckley 2020), redesigned to be delivered digitally during the pandemic, incorporated opportunities for networking and the ability to review and maintain access to TPD materials and their community of practice. Faculty attendees indicated these elements were high valued (ibid.)

Philipsen et al. (2019) also see lack of time as a barrier to participate in TPDs, although online flexible curricula are designed to help participants fit their studies around busy schedules (Allen and Seaman 2015). The authors of the paper also noted the importance of cost effectiveness. Gregory and Salmon’s (2013) TPD course in an Australian institution was delivered online with the idea of keeping costs low and increasing sustainability. This becomes even more critical in times of rapid shift to digital instruction, financial losses due to public disinvestment (Leins 2020), and the need for affordable scalable solutions post-pandemic.

Course-in-a-box (CIB), an online TPD for OBL initiative offered by a public research university in the Southern United States, includes categories and guidelines highlighted by Philipsen et al., such as support and feedback from learning technologists, flexibility and sustainability, and clear program goals and procedures. In this guided do-it-yourself course development service, faculty enroll in an online asynchronous training course, which has a set of goals and objectives, covers topics such as backwards design, accessibility and copyright, and best practices in assessment and student interactions. On completion of the training, faculty are given access to a course template, which they use to develop their online course. During course development, faculty are given the contact of eLearning support professionals who review their course at key development milestones. Philipsen et al. note that clear objectives and templates are crucial to a successful TPD experience for online teaching and learning. Indeed, since the inception of this TPD initiative in 2019, 673 faculty completed the training and 169 new online courses were developed (Smatresk 2020), which followed instructional design (Quality Matters 2018), accessibility (W3C 2018), and copyright and trademark compliance standards. The demand is growing and the program is revisited every 6 months to address opportunities for improvement and to keep it current. Relevance and evaluation also emerged in Philipsen et al.’s review as key components for a successful TPD program.

## Limitations and future suggestions

The authors selected research papers published between 2004 and 2015. In the fast-paced field of online and blended instruction, 5 years is a significant timeframe for technology to evolve and for teachers and students to adapt to new tools and pedagogies. Additionally, most quality online courses are designed and developed within 6–12 months (Li and Shearer 2005). This study was published before the COVID pandemic hit, revolutionizing how people live, study, and work globally (e.g., Arango 2020; Favale et al. 2020).

The paper focused mostly on the impact of TPD for OBL on teachers. Student-centered approaches to evaluating impact could be used in future research, investigating how the synthesized findings from Philipsen et al. (2019), forming their framework of important components of TPD for OBL, correlate to students’ learning and engagement. Investigating the impact of a TPD for OBL on the teachers’ own students may provide critical insights as the demographics and needs of students continue to evolve. Since the publication of the 15 studies reviewed by Philipsen and colleagues, there are more first-generation college students, with fewer financial means, and more students entering college underprepared (Gabriel 2018).

Finally, resiliency can be an important trait in delivering effective instruction during a pandemic (Kapasia et al. 2020; Zhu and Liu 2020). Merriam-Webster (n.d.) defines resiliency as “an ability to recover from or adjust easily to adversity or change”. Training on flexible and resilient teaching in online, hybrid flexible or ‘hyflex’ environments appears to enable institutions to maintain educational activities during a disruption (Glantz and Gamrat 2020). A future iteration of the meta-aggregate for TPD for OBL initiatives over the last 5 years (2016–2020 inclusive) could shed further light on the important components of TPD resiliency for OBL.
